# Las infecciones fúngicas: una amenaza creciente

**DOI:** 10.7705/biomedica.7214

**Published:** 2023-08-31

**Authors:** Beatriz L. Gómez, Patricia Escandón

**Affiliations:** * Grupo de Estudios en Microbiología Translacional y Enfermedades Emergentes (MICROS), Escuela de Medicina y Ciencias de la Salud, Universidad del Rosario, Bogotá, D.C., Colombia Universidad del Rosario Escuela de Medicina y Ciencias de la Salud Universidad del Rosario Bogotá, D.C. Colombia; ** Grupo de Microbiología, Instituto Nacional de Salud, Bogotá, D.C., Colombia Instituto Nacional de Salud Bogotá, D.C. Colombia

De todos los microorganismos patógenos para los humanos, históricamente los hongos han sido los menos estudiados y atendidos por los programas de salud pública a nivel nacional y mundial. La importancia médica, veterinaria y ecológica de las enfermedades fúngicas ha aumentado considerablemente en el pasado. La carga actual de las enfermedades micóticas en humanos supera varios millones de casos y se calculan más de 1,5 millones de muertes al año en todo el mundo, además de que el costo de su diagnóstico y tratamiento tiene un importante impacto económico y en la salud pública ^1^^-^^5^ (https://gaffi.org/).

Las causas de esta situación actual son complejas, pero la mayoría refleja una confluencia de actividades humanas que han resultado en avances significativos en la medicina moderna. Estas causas incluyen, entre otras: los efectos y dimensiones de la epidemia del VIH/sida como consecuencia de la difusión del virus de la inmunodeficiencia humana (VIH) alrededor del mundo y que ha dado como resultado un gran número de personas con inmunidad debilitada; la aparición de nuevos hongos patógenos, como *Candida auris*, que presenta un alto grado de resistencia a los antifúngicos disponibles y que, además, ha causado brotes en las unidades de cuidados intensivos ^6^, y factores globales como el incremento del fenómeno migratorio, los viajes, el cambio climático, los desastres naturales y los provocados por el hombre ^1^^,^^5^^,^^7^^-^^8^.

Todo esto se suma a que las infecciones causadas por hongos son difíciles de tratar porque tienden a ser crónicas, muchas de ellas difíciles de diagnosticar y de erradicar con los medicamentos antimicóticos disponibles actualmente. Por último, y no menos importante, las predicciones climáticas para el siglo XXI prevén un calentamiento global progresivo que reducirá el gradiente de temperatura de los mamíferos y el ambiente. Esto combinado con la capacidad de los hongos para adaptarse a temperaturas más altas aumenta la posibilidad de nuevas enfermedades fúngicas ^1^^,^^9^^-^^12^.

Antes de la pandemia por el virus SARS-CoV-2, la resistencia a los antibióticos figuraba como la prioridad mundial de la atención en salud. Algunos análisis, incluido el informe de O’Neill en 2022 ^13^, han pronosticado que las muertes debidas a infecciones bacterianas resistentes a medicamentos podrían eclipsar el número total de muertes por cáncer para el 2050. Aunque las infecciones fúngicas permanecen en la sombra de la conciencia pública, las muertes anuales totales atribuibles son similares o superan la mortalidad global por malaria, tuberculosis o VIH. El impacto de las infecciones fúngicas se ha visto exacerbado por el aumento constante de cepas y especies resistentes a los fármacos antifúngicos, lo que refleja su uso generalizado para la profilaxis y la terapia, y en el caso de la resistencia a los azoles en *Aspergillus* spp., se ha relacionado con el uso generalizado de antifúngicos en la agricultura ^14^^-^^19^.

Actualmente, en la práctica clínica solo se utilizan cuatro clases de medicamentos antimicóticos sistémicos: los azoles, las equinocandinas, las pirimidinas y los polienos, y solo unos pocos más están en desarrollo ^5^^,^^20^^-^^23^. Aunque los medicamentos antimicóticos existentes son efectivos, están asociados con muchos efectos adversos. La prescripción de estos medicamentos también requiere experiencia y sus interacciones farmacológicas con otros son particularmente comunes ^5^^,^^24^. Tales interacciones, junto con el requisito de ciclos prolongados de terapia, afectan aún más la seguridad y el pronóstico del paciente.

La pandemia por coronavirus (COVID-19) también se ha asociado con un aumento en la incidencia de infecciones fúngicas invasivas comórbidas. La aspergilosis, la mucormicosis y la candidemia fueron las infecciones fúngicas más frecuentemente asociadas con COVID-19, y se informaron con frecuencia con consecuencias devastadoras ^5^^,^^25^.

En el ámbito médico específicamente, los pacientes en tratamiento de cánceres con medicamentos que alteran la inmunidad, los que reciben agentes inmunosupresores para tratar enfermedades autoinmunitarias, los que son sometidos a procedimientos invasivos tales como cirugías o colocación de catéteres intravasculares, y aquellos que reciben de forma profiláctica antibióticos y antimicóticos que modifican el microbioma normal, se convierten en huéspedes propensos a padecer infecciones causadas por hongos. Estas infecciones fúngicas son más recalcitrantes a la terapia en comparación con la mayoría de las infecciones bacterianas. Por lo tanto, los pacientes requerirán tratamientos largos, lo que a su vez aumenta el riesgo de desarrollo de resistencia antifúngica.

A pesar de los avances, la terapia para la mayoría de las enfermedades fúngicas invasivas sigue siendo insatisfactoria dada su alta morbilidad y mortalidad ^1^^,^^5^^,^^14^, como en los casos de la aspergilosis, la candidiasis y la criptococosis, que presentan una alta mortalidad -incluso con el tratamiento adecuado- y, a menudo, son incurables en huéspedes con inmunidad deteriorada ^1^.

A diferencia de las enfermedades bacterianas y virales, las infecciones fúngicas invasoras rara vez son transmisibles y esto ha llevado a un menor interés de las autoridades de salud pública en cuanto a su vigilancia, por lo que hay poca información sobre la incidencia y la prevalencia de estas micosis.

Es importante también mencionar que se pueden presentar “brotes” o “epidemias” causados por hongos y, cuando ocurren, tienden a reflejar una de tres situaciones:

1. aumento de la prevalencia de huéspedes con inmunidad comprometida vulnerables a estas infecciones;
2. exposiciones a un gran inóculo, como los brotes de histoplasmosis tras la exposición al agente causal en actividades como la tala de árboles en zonas endémicas, visitas a cuevas o remoción de material contaminado ^26^^-^^27^, y
3. causas iatrogénicas como, por ejemplo, el brote de meningitis fúngica por *Exserohilum rostratum* después de la aplicación de soluciones contaminadas de esteroides ^28^ o el brote causado por *Sarocladium kiliense* luego de la administración de un medicamento antiemético a pacientes oncológicos ^29^.

Considerando todo lo mencionado anteriormente, además de la importancia de establecer criterios que definan una especie fúngica y alcanzar una construcción filogenética más o menos estable que permita entender los cambios taxonómicos de una manera práctica ^30^^,^^31^, tenemos un panorama actual en el que las enfermedades fúngicas son cada vez más prevalentes en ambos, humanos y animales, con opciones de tratamiento insatisfactorias para los primeros y pocas o ninguna para los segundos. Es muy probable que, en los próximos años, el problema de las infecciones fúngicas invasivas tienda a crecer y la humanidad se enfrente a nuevas amenazas con especies de hongos que actualmente no las representan.

A pesar de esta situación compleja causada por el incremento de las enfermedades fúngicas en humanos vulnerables y en diversos ecosistemas, el reino de los hongos tiende a ignorarse si se compara con las bacterias, los virus y los parásitos. La revista *Nature Microbiology* lo señaló recientemente en el editorial titulado “Stop neglecting fungi” ^32^.

Sin embargo, también es importante mencionar y reconocer que tenemos un progreso importante gracias a instituciones como el *Global Action Fund for Fungal Infections* (GAFFI) (https://gaffi.org/), que desde el año 2013 ha sido una de las voces a nivel mundial sobre las enfermedades fúngicas en términos de salud pública y estima que, con un mejor diagnóstico y tratamiento disponible, el número de muertes por enfermedades por hongos podría disminuir a menos de 750.000 por año.

Para lograr esto, GAFFI se encuentra trabajando con sectores de la academia, la industria y los entes de salud pública en políticas que comprenden cinco áreas temáticas:

1. minimizar las muertes por sida debido a enfermedades fúngicas,
2. diagnosticar correctamente la enfermedad pulmonar fúngica similar a la tuberculosis,
3. crear conciencia en los organismos de salud pública regionales y mundiales sobre las enfermedades tropicales desatendidas, por hongos,
4. ayudar a ampliar los servicios de laboratorio para el diagnóstico de enfermedades fúngicas mediante educación y transferencia de tecnología y
5. atender la resistencia antifúngica como problema global urgente (https://gaffi.org/).

Estos esfuerzos se unen también a iniciativas por la academia y un ejemplo es el de investigadores en el Reino Unido, que también ha sentado un precedente importante, con estrategias que reconocen la importancia de la financiación de proyectos que contribuyan al avance de la micología médica ^33^ y de otras agencias en los Estados Unidos, como los *Centers for Disease Control and Prevention* (CDC), líder mundial en el área de la salud pública. Es gracias a todos estos esfuerzos y al de muchas otras instituciones en los distintos países, que estamos logrando poco a poco avances importantes.

Es de resaltar que, en el 2017, la Organización Mundial de la Salud (OMS) elaboró su primera lista de agentes patógenos bacterianos prioritarios en el contexto del aumento de la resistencia a los antibióticos para ayudar a impulsar la acción mundial en la investigación y el desarrollo de nuevos tratamientos ^34^. Inspirándose en este documento, la OMS ha desarrollado y publicado recientemente la primera lista de agentes fúngicos prioritarios ^5^. Este documento representa el primer esfuerzo mundial para priorizar sistemáticamente los agentes patógenos fúngicos, teniendo en cuenta sus necesidades insatisfechas de investigación y desarrollo, y su importancia percibida para la salud pública.

Este documento de la OMS tiene como objetivo enfocar e impulsar más investigaciones e intervenciones políticas para fortalecer la respuesta global a las infecciones fúngicas y a la resistencia a los antifúngicos ^5^. Propone tres áreas principales de acción:

1. fortalecimiento de la capacidad y la vigilancia de los laboratorios;
2. inversiones sostenibles en investigación, desarrollo e innovación; y
3. intervenciones de salud pública.

Los agentes patógenos se clasificaron en tres grupos de prioridad (crítica, alta y media). El grupo crítico incluye *Cryptococcus neoformans*, *Candida auris*, *Aspergillus fumigatus* y *Candida albicans*. El grupo de prioridad alta incluye *Nakaseomyces glabrata* (*Candida glabrata*), *Histoplasma* spp., agentes causantes de micetoma, mucorales, *Fusarium* spp., *Candida tropicalis* y *Candida parapsilosis*; y los agentes patógenos del grupo de prioridad media son *Scedosporium* spp., *Lomentospora prolificans*, *Coccidioides* spp., *Pichia kudriavzeveii* (*Candida krusei*), *Cryptococcus gattii*, *Talaromyces marneffei*, *Pneumocystis jirovecii* y *Paracoccidioides* spp.

Este documento propone acciones y estrategias para formuladores de políticas, profesionales de la salud pública y otras partes interesadas, con el objetivo de mejorar la respuesta general a los patógenos fúngicos prioritarios, incluyendo la prevención y el desarrollo de resistencia a medicamentos antifúngicos ^5^.

En consecuencia, existe una necesidad apremiante de más investigación en los diferentes campos que abarca la micología médica. Se necesitan mejores herramientas diagnósticas, nuevos medicamentos antimicóticos, más seguros y efectivos, una mejor comprensión de la interacción del huésped con el hongo y de la respuesta inmunitaria, y un consenso en la cambiante taxonomía de los hongos. Todo esto con el fin de aprovechar al máximo todas las herramientas modernas para llevar a cabo nuevos estudios y establecer redes interactivas entre los diferentes grupos de investigación, la industria farmacéutica y las entidades de salud pública.

El objetivo de este suplemento es contribuir a divulgar temas relevantes y estratégicos en el campo de la micología médica, con revisiones de tema hechas por expertos, presentaciones de casos y artículos originales. A través de estas contribuciones se espera resaltar la importancia de investigar en el campo de la micología médica desde la ciencia básica y desde la clínica.

Este suplemento está dedicado a la memoria y al gran legado de la doctora Ángela Restrepo Moreno, una de las principales figuras de la ciencia en Colombia, cuyas contribuciones a la Micología Médica la convirtieron en una pionera y un referente en Latinoamérica y a nivel mundial. Las editoras de este suplemento agradecen de una manera muy especial a la revista *Biomédica* por este espacio, y a todos los colegas, en Colombia, en los distintos países en Latinoamérica y en los Estados Unidos, por sus importantes contribuciones.

## References

[1] Casadevall A. (2018). Fungal Diseases in the 21st Century: The near and far horizons. Pathog Immun.

[2] Bongomin F, Gago S, Oladele RO, Denning DW. (2017). Global and multi-national prevalence of fungal diseases - estimate precision. J Fungi (Basel).

[3] Brown GD, Denning DW, Gow NA, Levitz SM, Netea MG, White TC. (2012). Hidden killers: Human fungal infections. Sci Transl Med.

[4] Rodrigues ML, Albuquerque PC. (2018). Searching for a change: The need for increased support for public health and research on fungal diseases. PLoS Negl Trop Dis.

[5] World Health Organization (2022). WHO fungal priority pathogens list to guide research, development and public health action.

[6] Lockhart SR, Etienne KA, Vallabhaneni S, Farooqi J, Chowdhary A, Govender NP (2017). Simultaneous emergence of multidrug-resistant Candida auris on 3 continents confirmed by whole-genome sequencing and epidemiological analyses. Clin Infect Dis.

[7] Smith DFQ, Casadevall A. (2022). Disaster microbiology-a new field of study. mBio.

[8] Casadevall A. (2017). Don’t forget the fungi when considering global catastrophic biorisks. Health Secur.

[9] Garcia-Solache MA, Casadevall A. (2010). Global warming will bring new fungal diseases for mammals. MBio.

[10] Wu X, Lu Y, Zhou S, Chen L, Xu B. (2016). Impact of climate change on human infectious diseases: Empirical evidence and human adaptation. Environ Int.

[11] Nnadi NE, Carter DA. (2021). Climate change and the emergence of fungal pathogens. PLoS Pathog.

[12] Frías-de-Leon MG, Brunner-Mendoza C, Reyes-Montes M del R, Duarte-Escalante E (2022). The impact of climate change on fungal diseases.

[13] O’Neill J. (2022). Tackling drug-resistant infections globally: Final report and recommendations. Government of the United Kingdom.

[14] Gow NAR, Johnson C, Berman J, Coste AT, Cuomo CA, Perlin DS (2022). The importance of antimicrobial resistance in medical mycology. Nat Commun.

[15] Denning DW. (2022). Antifungal drug resistance: an update. Eur J Hosp Pharm Sci Pract.

[16] Reddy GKK, Padmavathi AR, Nancharaiah YV. (2022). Fungal infections: Pathogenesis, antifungals and alternate treatment approaches. Curr Res Microb Sci.

[17] Pfaller MA. (2012). Antifungal drug resistance: mechanisms, epidemiology, and consequences for treatment. Am J Med.

[18] Rhodes J, Abdolrasouli A, Dunne K, Sewell TR, Zhang Y, Ballard E (2022). opulation genomics confirms acquisition of drug-resistant Aspergillus fumigatus infection by humans from the environment. Nat Microbiol.

[19] Zhou D, Korfanty GA, Mo M, Wang R, Li X, Li H (2021). Extensive genetic diversity and widespread azole resistance in greenhouse populations of Aspergillus fumigatus in Yunnan, China. mSphere.

[20] Osherov N, Kontoyiannis DP. (2017). The anti-Aspergillus drug pipeline: Is the glass half full or empty?. Med Mycol.

[21] Perfect JR. (2017). The antifungal pipeline: a reality check. Nat Rev Drug Discov.

[22] Hoenigl M, Sprute R, Egger M, Arastehfar A, Cornely OA, Krause R (2021). The antifungal pipeline: Fosmanogepix, ibrexafungerp, olorofim, opelconazole, and rezafungin. Drugs.

[23] Denning DW, Bromley MJ. (2015). Infectious disease. How to bolster the antifungal pipeline. Science.

[24] Niazi-Ali S, Atherton GT, Walczak M, Denning DW. (2021). Drug-drug interaction database for safe prescribing of systemic antifungal agents. Ther Adv Infectious Dis.

[25] Raut A, Huy NT. (2021). Rising incidence of mucormycosis in patients with COVID-19: another challenge for India amidst the second wave?. Lancet Respir Med.

[26] Deepe GS. (2018). Outbreaks of histoplasmosis: The spores set sail. PLoS Pathog.

[27] Armstrong PA, Beard JD, Bonilla L, Arboleda N, Lindsley MD, Chae SR (2018). Outbreak of severe histoplasmosis among tunnel workers-Dominican Republic, 2015. Clin Infect Dis.

[28] Andes D, Casadevall A. (2013). Insights into fungal pathogenesis from the iatrogenic epidemic of Exserohilum rostratum fungal meningitis. Fungal Genet Biol.

[29] Etienne KA, Roe CC, Smith RM, Vallabhaneni S, Duarte C, Escadón P (2016). Wholegenome sequencing to determine origin of multinational outbreak of Sarocladium kiliense bloodstream infections. Emerg Infect Dis.

[30] Naranjo-Ortiz MA, Gabaldón T. (2019). Fungal evolution: Diversity, taxonomy and phylogeny of the fungi. Biol Rev Camb Philos Soc.

[31] Spatafora JW, Aime MC, Grigoriev IV, Martin F, Stajich JE, Blackwell M. (2017). The fungal tree of life: From molecular systematics to genome-scale phylogenies.. Microbiol Spectr.

[32] No authors listed (2017). Stop neglecting fungi. Nat Microbiol.

[33] Gow NAR, Amin T, McArdle K, Brown AJP, Brown GD, Warris A (2018). Trends Microbiol.

[34] World Health Organization (2017). Prioritization of pathogens to guide discovery, research and development of new antibiotics for drug-resistant bacterial infections, including tuberculosis.

